# Supplementary material for: Polycistronic Artificial microRNA-Mediated Resistance to Cucumber Green Mottle Mosaic Virus in Cucumber
Source: Int J Mol Sci. 2021 Nov 12;22(22):12237. doi: 10.3390/ijms222212237 (PMC8620374; doi:10.3390/ijms222212237)
Supplement: Supplementary file 1 [file ijms-22-12237-s001.zip › ijms-1443447-supplementary.pdf]

Supplementary Material

Table S1. The sequence information related with artificial miRNA(amiRNA)

amiRNA	Sequence of amiRNA (5’-3’)	Primer	Sequence of Primer (5’-3’)	Target gene	Backbone
amiR1-Rep	TAAAATCATAGGAACACTCAT	Primer_ 1	CACCAAACAAAACCTCACAATGAGAGAGTCCCTGT	CGMMV Rep	AMIR171vl
		Primer_2	GTGTGTGGTCAGGTAAGAAATGAGTGTTCCTATGATTTTAGAACAGGGACTCTCTCA		
		Primer_3	TCTTACCTGACCACACACGTAGATATACATTATTCTCTCATGATTA		
		Primer_4	TTATTCTCTCATGATTAAATGAGTGTTACTATGATCTTAGATCAGTACTCTCTCGTCAA		
		Primer_5	ATGTGTGATTACTTACTTTGGTTGACGAGAGAGTACTGA		
amiR4-MP	TTTITGTAAGATCAACAGCAC	Primer_2	GTGTGTGGTCAGGTAAGAAGTGCTGTTGATCTTACAAAAAGAACAGGGACTCTCTCA	CGMMV MP	
		Primer_4	TTATTCTCTCATGATTAAGTGCTGTTGCTCTTACACAAAGATCAGTACTCTCTCGTCAA		
amiR2-Rep	TGTATAATGCAAAATAGACTC	Primer_1	CACCAAACCTCCACCAACAATGGGTGAGATTCTCCAT	CGMMV Rep	AMIR164vl
		Primer_2	ATTTCGTGTTTGTTGGTTAGAGTCTATTTTGCATTATACAGAATGGAGAATCTCACC		
		Primer_3	AACCAACAAACACGAAATCCGTCACATTTGCTTATT		
		Primer_4	CGTCACATTTGCTTATTAGAGTCTATTCTGCATTACACAGAATGAGCACTTCACCCCAA		
		Primer_5	AGTTCTGTTGGGTAATGGTATTGGGGTGAAGTGCTCAT		
amiR5-MP	TAAATAAAGACCAAGGATCTC	Primer_2	ATTTCGTGTTTGTTGGTTAGAGATCCTTGGTCTTTATTTAGAATGGAGAATCTCACC	CGMMV MP	
		Primer_4	CGTCACATTTGCTTATTAGAGATCCTTAGTCTTTACTTAGAATGAGCACTTCACCCCAA		
amiR3-Rep	TTTTATCAGACAITAACTCGT	Primer_1	CACCAACCTGAACTAAACACAAGAGAAACGCAAAGAA	CGMMV Rep	AMIR156vl
		Primer_2	TATGCAAATTGCCTTTGTAACGAGTTAATGTCTGATAAAAGATTCTTTGCGTTTCTC		
		Primer_3	ACAAAGGCAATTTGCATATCATTGCACTTGCTTCTCTTGC		
		Primer_4	GCACTTGCTTCTCTTGCAACGAGTTAACGTCTGATCAAAGATTCCGGTGCTGATCTCTT		
		Primer_5	GTGGTTAATGGTTTTGTTGAAAGAGATCAGCACCGGAA		
amiR6-CP	TATAAGAAGCACTAAACGCAA	Primer_2	TATGCAAATTGCCTTTGTATTGCGTTTAGTGCTTCTTATAGATTCTTTGCGTTTCTC	CGMMV CP	
		Primer_4	GCACTTGCTTCTCTTGCATTGCGTTTAATGCTTCTCATAGATTCCGGTGCTGATCTCTT		

Table S2. Probe sequence and modification used in northern blot

amiRNA	Sequence of amiRNA (5’-3’)	5’ modification	3’ modification	Others
amiR1-probe	aTgaGtgTtcCtaTgatttta			
amiR2-probe	gAgtCtaTttTgcAttataca			
amiR3-probe	aCgaGttAatGtcTgataaaa			
amiR4-probe	gTgcTgtTgaTctTacaaaaa	5’ - Digoxin	3’ - Digoxin	Capital letters are decorated with LNA
amiR5-probe	gAgaTccTtgGtcTttattta			
amiR6-probe	tTgcGttTagTgcTtcttata			
amiR-GUS probe	tCggCaaAgtGtgGgtcaata			
U6-probe	cgatttgtgcgtgtcatccttg		3’ - Digoxin	

Table S3. Names and sequences of oligonucleotides used in this study

Oligonucleotide	Sequence (5’-3’)	Use
amiR1-Rep F	CGGGTAAAATCATAGGAACACTCAT	
amiR2-Rep F	GCCCCTGTATAATGCAAAATAGACTC	
amiR3-Rep F	AGCCCTTTTATCAGACATTAACTCGT	qRT-PCR amplification of amiRNA transcript
amiR4-MP F	AGGGTTTTTGTAAGATCAACAGCAC	
amiR5-MP F	CGGGTAAATAAAGACCAAGGATCTC	
amiR6-CP F	CGGGTATAAGAAGCACTAAACGC	
CGMMV-CP F	ACAGCCGCTAGGGCTGAGATA	qRT-PCR amplification of CGMMV CP transcript
CGMMV-CP R	CCAATGAGCAAACCGTTCGAT	
AJD21	CCCCTCACCACAGAGTCTGC	qRT-PCR amplification of *Nicotiana benthamiana* 60s ribosomal protein L25 transcript
AJD22	AAGGGTGTTGTTGTCCTCAATCTT	
EF-1α F	ACTGGTGGTTTTGAGGCTGGT	qRT-PCR amplification of cucumber Elongation Factor 1 alpha (eEF1) transcript
EF-1α R	CTTGGAGTATTTGGGTGTGGT	
LF2096	CACTGACGTAAGGGATGACGCA	PCR amplification of pEG100-amiRNA
LF2097	GGATCTGAGCTACACATGCTCAGGT	

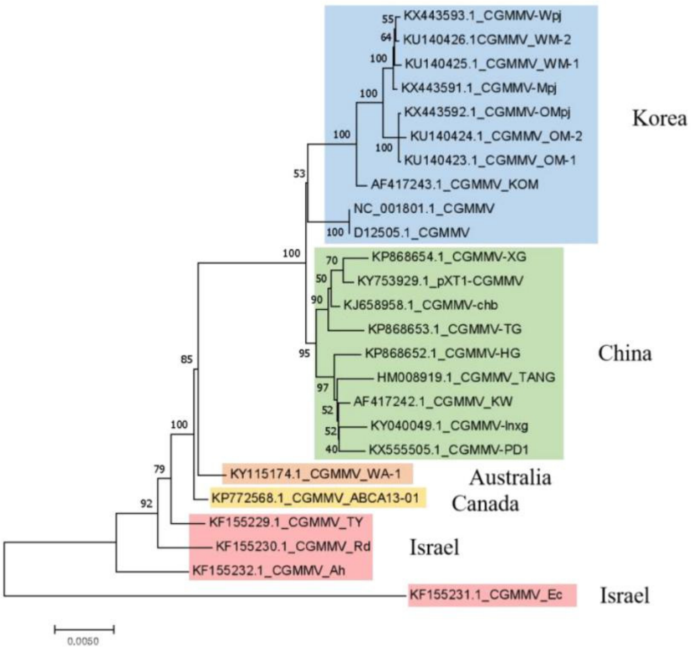

Figure S1. Phylogenetic tree based on the alignment of CGMMV amino acid sequences deduced from 25 CGMMV isolates using the Muscle algorithm within MEGA6 software. The tree was drawn to scale, with branch lengths measured as the number of amino acid substitutions per site (bar in the figure). The size of the bar represents 0.005 amino acid substitutions, indicating a high level of conservation of CGMMV amino acid sequences. Bootstrap values are indicated as the percentages shown at the nodes. The clade background colors identify the main clades. The largest blue and green clades represent East Asian isolates from Korea and China. The red clade contains isolates from Israel, and a single isolate, KP772568, from Canada is shaded yellow. Another isolate (KY115174.1) has been shown to be found in Australia. CGMMV, cucumber green mottle mosaic virus.

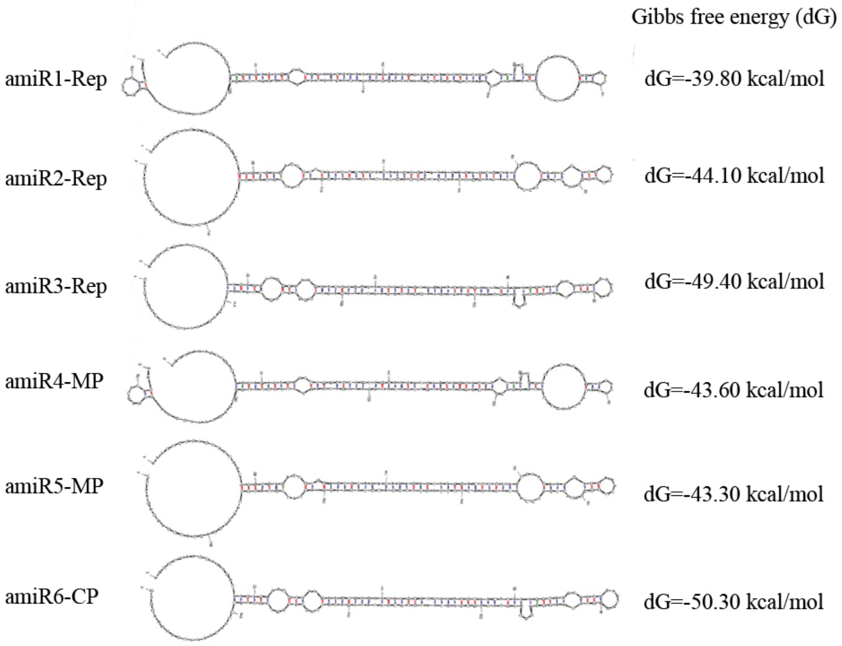

Figure S2. Predicted secondary structures of six CGMMV amiRNA precursors using mFold software. amiRNAs derived from precursors are designed to cleave CGMMV replicase-, movement-, or coat protein-encoding RNAs. The stability of the amiRNA precursors is indicated by the Gibbs free energy (dG) for each secondary structure. amiRNA, artificial microRNA.

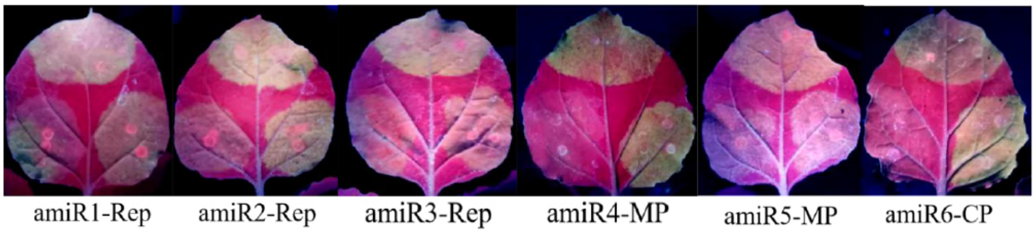

Figure S3. Specific detection of silencing targets of amiRNAs. The pGFPamiR target sensor constructs were co-infiltrated into the left half of *Nicotiana benthamiana* leaves with the mismatched amiRNA constructs, and the particular amiRNA constructs were co-infiltrated into the right half of *N. benthamiana* leaves. As a control, the original pEG100-miR171 and either of the pGFPamiR target sensor constructs were co-infiltrated. Three biological repeats of the transient assays displayed similar results. Rep, replicase; MP, movement protein; CP, coat protein.

(A)

CGMMV t gcc gta gaa gga
E

CGMMV^{E480G} t gcc gta gga gga
G

(B)

amiR2 Target	aag agt cta ctt tgc att ata ctc
	K C L L C I I L
CGMMV Res.	aag agt cta cta tgt atc atc ctc
amiR4 Target	cgt ggt gtt gat ctt aca aaa cac
	R G V D L T K H
CGMMV Res.	cgt ggt gtt gac cta act aag cat
amiR6 Target	tat tgc gtt tag tgc ttc tta tgt
	Y C V * C F L C
CGMMV Res.	tat tgc gtt taa tgt ttt ttg tgt

Figure S4. Base sequence mutation in CGMMV. **(A)** Mutation in amino acid sequence at position 480 of CGMMV from glutamic acid (E) to glycine (G). **(B)** At least five synonymous mutations (contain original mismatch sites) in the target regions of amiRNAs, where bases that differ from those of the wild-type virus isolate, were observed and are indicated in red.

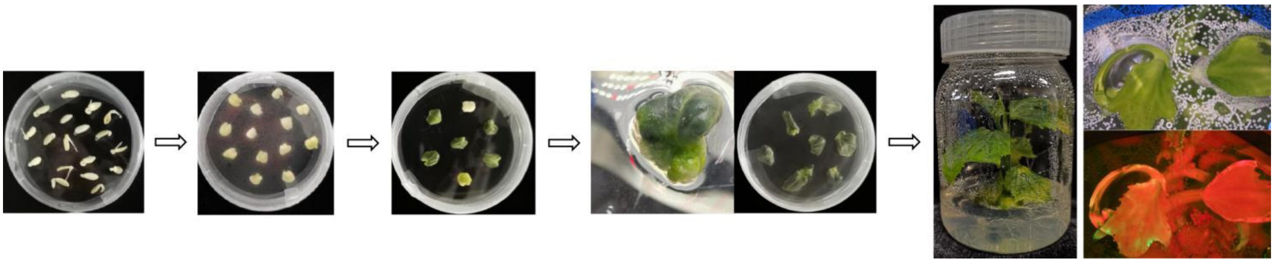

Figure S5. Flow chart depicting the construction of transgenic cucumber expressing amiR246. Positive transgenic plants were screened for based on the expression of green fluorescent protein co-expressed with amiRNA.

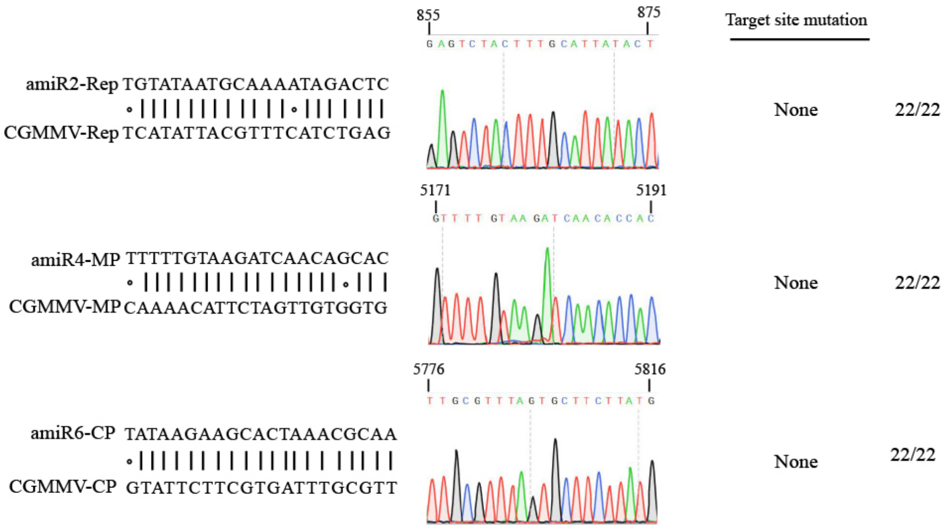

Figure S6. TS analysis in CGMMV-infected amiR-CGMMV lines. Electropherograms derived from sequencing CGMMV progeny, including the TS region only. TS, target sites.
